# Antecedents of teenage pregnancy from a 14-year follow-up study using data linkage

**DOI:** 10.1186/1471-2458-10-63

**Published:** 2010-02-11

**Authors:** Jennifer Gaudie, Francis Mitrou, David Lawrence, Fiona J Stanley, Sven R Silburn, Stephen R Zubrick

**Affiliations:** 1 Telethon Institute for Child Health Research, PO Box 855, West Perth, WA 6872, Australia; 2 Centre for Developmental Health, Curtin Health Innovation Research Institute, Curtin University of Technology, Perth, Western Australia

## Abstract

**Background:**

Many western nations continue to have high rates of teenage pregnancies and births, which can result in adverse outcomes for both mother and child. This study identified possible antecedents of teenage pregnancy using linked data from administrative sources to create a 14-year follow-up from a cross-sectional survey.

**Methods:**

Data were drawn from two sources - the 1993 Western Australian Child Health Survey (WACHS), a population-based representative sample of 2,736 children aged 4 to 16 years (1,374 girls); and administrative data relating to all their subsequent births and hospital admissions. We used weighted population estimates to examine differences between rates for teenage pregnancy, motherhood and abortion. We used Cox proportional hazards regression to model risk for teenage pregnancy.

**Results:**

There were 155 girls aged less than 20 years at the time of their first recorded pregnancy. Teenage pregnancy was significantly associated with: family type; highest school year completed by primary carer; combined carer income; whether the primary carer was a smoker; and whether the girl herself displayed aggressive and delinquent behaviours. An age-interaction analysis on the association with aggressive and delinquent behaviours found that while girls with aggressive and delinquent behaviours who were older at the time of the survey were at highest risk of teenage pregnancy, there was elevated risk for future teenage pregnancy across all ages.

**Conclusions:**

Our findings suggest that interventions to reduce teenage pregnancy rates could be introduced during primary school years, including those that are focused on the prevention and management of aggressive and delinquent behaviour.

## Background

Australia has a relatively high teenage pregnancy rate among western countries at 19.8 births per 1,000 women aged 15-19 years [1,2]. Of 30 OECD countries, Australia ranks 11th highest in teenage birth rate [3]. There are three main reasons why reducing the incidence of teenage pregnancy should be given a high priority. Firstly, teenage pregnancies are associated with increased medical risks during pregnancy and poorer birth outcomes for their babies [4-8]. Secondly, teenage mothers have poorer life-course outcomes in adulthood such as a higher risk of: dependence on welfare; being a sole parent; being a smoker; and having a lower socio-economic status [9-11]. Finally, the children of teenage mothers are more likely to be socio-economically disadvantaged, and have the potential to repeat the cycle of teenage pregnancies [12-14].

There is ongoing debate as to whether the adverse medical, social, educational and economic outcomes associated with teenage pregnancy are due to intrinsic risks of pregnancy and childbirth in the teenage years, or are associated with the social, economic and environmental circumstances of teenagers and their offspring [5]. Nonetheless it is widely accepted that even for teenagers who live in poverty and disadvantage, teenage pregnancy compounds the handicaps associated with social disadvantage, and initiatives aimed at reducing teenage pregnancy rates have the potential to expand the options and choices available for young people to overcome disadvantaged backgrounds [15-18]. With greater understanding of the antecedents of teenage pregnancy it may be possible to develop more effective interventions with the aim of reducing teenage pregnancy rates.

A range of antecedents of teenage pregnancy have been identified in the literature. Among the strongest and most persistent associations are measures of social disadvantage, inequality and social exclusion [10,12,19]. A pattern of intergenerational transfer has also been observed with daughters of teenage mothers being at high risk to become teenage mothers themselves [14], which has been associated with the transfer of disadvantage between generations [20], and with an association with childhood aggressive behaviours [21]. Aggressive and antisocial behaviours have also been independently associated with teenage pregnancy [19]. Dysfunctional family relationships, family breakdown and sole parent family structures are associated with higher rates of teenage pregnancy [19,20].

In this study we used a large, well-defined cross-sectional population sample of children which was subsequently linked to health system data spanning 14 years. The Western Australian Child Health Survey (WACHS) featured a framework of variables based on a social-ecology model allowing for extensive family-based demographic, psychosocial, health, educational and direct measures of both parents and survey children [22,23]. We hypothesised that there are factors in early life that are predictors of teenage pregnancy. As the girls in the WACHS were aged 4 to 16 years at the time of the survey, the factors measured in the survey were collected across a range of life stages. Using administrative record linkage to ascertain occurrence of births or abortions, we set out to: a) quantify the association of known risk factors for teenage pregnancy within our WA sample; b) investigate if other factors within the social-ecology framework were independently associated with teenage pregnancy outcomes; and c) investigate whether the observed associations changed with the age of the girl at the time when the risk factors were assessed.

## Methods

### The 1993 Western Australian Child Health Survey (WACHS)

The subjects for this data linkage study were the 1,374 girls sampled in the 1993 WA Child Health Survey, a population-based state-wide representative study of 2,736 children aged 4 to 16 years in Western Australia [24]. Dwellings were randomly selected and participation in the survey was voluntary. Overall 82% of contacted households participated in the survey. All survey data were gathered under appropriate institutional ethics clearance and supervision. Data collection took place from early July to the end of September 1993. For consenting families, data were gathered from personal interviews, forms completed by the family (by the primary carer and by children aged 12 to 16 years) and school administered forms (by the school Principal and by the school teacher with the best knowledge of the child). Aboriginal children living in country areas were excluded from the survey.

The personal interview comprised three main forms -- the Household Record Form (which recorded family member details and basic demographic information), the Family Background Questionnaire (which collected information about the primary and secondary carer of each survey child) and the Child Health Questionnaire (which recorded details of the child's physical and mental health).

Two forms were self-administered by the primary carer, the Family Health and Activity Questionnaire (collected details on parental health, relationships and neighbourhood environment) and the Child Behavior Checklist (CBCL). The CBCL is a 112-item instrument which was used to estimate mental health morbidity among children in the 1993 survey. The CBCL is one of the products from the Achenbach System of Empirically Based Assessment (ASEBA) [25]. The CBCL allows the calculation of an overall summary measure (Total Problem Behaviour) and eight individual syndrome scores: delinquent behaviour, aggressive behaviour, withdrawn, anxious/depressed, somatic complaints, social problems, thought problems and attention problems. These scores are standardised as T-scores. The CBCL was administered to both the child's primary carer and their teacher. The respective T-scores were combined to create a categorical variable measuring overall mental health morbidity [26].

### The Western Australian Data Linkage System (WADLS)

Subjects were followed up using linkage to administrative data obtained from the births and midwives collections, and the hospital morbidity data system, prepared by the Western Australian Data Linkage Unit (WADLU) using the Western Australian Data Linkage System (WADLS). This enables linkage of total population data for epidemiological and health services research. Survey data were linked with health service utilisation data collected between the time of the survey (i.e. 1993) and 31 December 2007. We also obtained birth records for the survey children. At the end of the follow-up (i.e. December 2007) the survey children were aged between 18 and 31 years.

At the time of the survey, carers and young people aged 12 years and over gave consent for access to medical and school records as part of the consent to participate in the study. As the WADLS did not exist in 1993 and no time period was specified in the consent arrangements in the survey, it was assumed that participants in the survey did not give specific consent to a record linkage study being undertaken 15 years later. This study was undertaken under the provisions for record linkage where consent is not obtained. Ethical approval for this record linkage study was granted by the Human Research Ethics Committee of Curtin University of Technology and the Human Research Ethics Committee of the Western Australian Department of Health. To preserve the privacy and confidentiality of the information collected from participants in the WACHS, a strict protocol was established and followed to ensure that all research was conducted on de-identified files and that no researcher would be in a position to access identifying information for any study participant. The WACHS data custodians provided the list of names and addresses of survey participants to the WADLU, along with the mother's maiden name and the hospital where the child was born to increase the accuracy of the data linkage. Once the data were linked, these identifying variables were replaced with a unique encrypted record linkage key. This key was used to join the de-identified WACHS file with the birth and abortion outcomes. This de-identified, anonymised file was then sent to the analysts to complete the study. No-one associated with the study had access to both the named WACHS data and the de-identified linked file, which was maintained on a separate secure server at a separate site.

### Outcome measurement

For the purposes of this analysis, a teenage pregnancy was identified by either a record of a live birth or abortion of a pregnancy that was reported from WA public and private hospitals between 1993 and 2007. Miscarriages were not included as they were not available on the linked data. Three outcomes were examined in our analyses: becoming pregnant as a teenager; becoming a teenage mother; and having a pregnancy aborted as a teenager. A girl was classified as a teenage mother if her first pregnancy resulting in a live birth occurred before she had reached 20 years of age. A girl was classified as having an abortion as a teenager if her first abortion occurred before she had reached 19 years and 6 months (due to the expected age of mother at time of delivery had the pregnancy continued, and that most abortions are carried out in the first trimester). We calculated the proportion of girls from the WACHS who had each of these outcomes overall, and then compared these proportions by variables from the WACHS. Individual, primary carer, family and community level characteristics were examined as potential correlates of these outcomes.

Individual characteristics of the teenager included her combined parent and teacher CBCL score, her birth weight, whether she was breastfed, the type of school she had attended (i.e. government, catholic and independent), how well the girl did at school (as reported by the primary carer and teacher) and whether she needed professional help for her emotional or behavioural problems (as reported by the primary carer). Characteristics of the primary carer (usually the girl's mother) included whether they had been a smoker, whether they themselves had been a teenage mother, their highest school year completed, the level of importance of religion to them, whether they were in receipt of government benefits, whether they had ever been treated for an emotional or mental health problem, their marital status, their level of satisfaction with their daughter's progress in her education and learning skills, and whether they had ever been arrested [27].

Family level characteristics included family type (original, step/blended or sole-parent), size of the family, combined carer income, whether the family lived in a metropolitan or non-metropolitan area, and the level of family functioning (as measured by the McMaster Family Assessment Device, FAD) [28]. Combined carer income, measured in 1993 Australian dollars, was defined as low (less than $600 per week), medium ($600 to $1100 per week) or high (over $1100 per week), which represented terciles of household income in 1993. One community level characteristic used as a potential predictor was the Socio-Economic Indexes for Areas (SEIFA). Based on Census information, these indexes provide a measure of disadvantage and can be used to assess socio-economic conditions by geographical areas [29].

### Weighting and estimation procedures

The WACHS was a stratified, clustered representative probability sample [24]. Western Australia was stratified into 6 geographic regions. Within each stratum, the sample was selected in two stages. The first stage entailed selection of census collection districts, and the second stage was selection of families. Within selected families all eligible children (aged 4-16 years) were included in the survey. Weights were employed to account for selection probabilities and correct for potential non-response biases, with post-stratification by age, sex, family size and geographic area. Because of the stratification, clustering and weighting, the statistical methods used needed to account for these aspects of the survey design. Proportions were estimated using the survey weights to produce unbiased population estimates. Variances and confidence intervals on these estimates were produced using the ultimate cluster variance estimation technique [30]. This accounted for the clustered nature of the original survey sample. Full details of the survey methodology, weighting and estimation procedures have been described elsewhere [24].

### Proportional hazards regression analysis

The association between factors collected in the WACHS and teenage pregnancy was assessed using multivariate proportional hazards regression. To account for the clustered nature of the survey sample, the Lin and Wei robust sandwich estimate of the variance-covariance matrix was employed [31]. Proportional hazards regression was chosen because although all girls in the study had the same length of follow-up time, not all girls had passed through the teenage years by the end of follow-up. However, as the age of the children in the WACHS ranged from 4-16 years in 1993, the independent variables in the model were measured at different life stages for different children. To examine whether age at time of the WACHS affected the association with aggressive and delinquent behaviour and risk for teenage pregnancy, we fitted an age interaction model using age as a continuous variable. As proportional hazards regression models the log of the hazard ratio, it is generally not appropriate to assume that the association with a continuous variable will be linear (as this would imply an exponential relationship with age on the hazard ratio scale). As there were no theoretical grounds to hypothesise any particular shape for this relationship, we fitted a non-parametric spline curve using generalised additive models [32]. All statistical analyses except for the proportional hazards regression were undertaken using SAS (version 9; SAS Institute, Cary, NC). The proportional hazards regression model was fitted using Hastie and Tibshirani's GAIM software [32] within Stata (version 10; StataCorp, College Station, TX).

We evaluated the power of the study based on the sample design and expected frequency of the outcome. For a predictor with prevalence of 10%, the study would have about 80% power to detect a hazard ratio of 1.7, or 60% power to detect a hazard ratio of 1.5. Stronger power is associated with larger effect sizes or more prevalent risk factors. The study would only have modest power to detect associations with rarely occurring risk factors or risk factors associated with small effect sizes.

## Results

There were 1,374 girls in the WACHS, of whom 381 had at least one recorded pregnancy on the linked dataset during the follow-up period. Of these, 155 were aged less than 20 years at the time of their first pregnancy. Of these teenagers, 72 had an abortion and 94 became mothers (11 teenagers had both). Table 1 shows the weighted proportions of girls from the 1993 WACHS by factors collected in the survey. Table 2 reports the weighted proportions of girls from the original survey who: i) went on to become pregnant as a teenager (10.5%); ii) became teenage mothers (6.9%); and iii) went on to have an abortion as a teenager (4.2%), cross-classified by variables collected in the 1993 WACHS. Each weighted proportion is presented with 95% confidence intervals.

**Table 1:** Characteristics of Western Australian girls aged 4-16 years in 1993

Characteristic	Proportion (%)	95% CI
Family type		
Original	73.2	69.0 - 77.0
Step/blended	9.8	2.6 - 12.2
Sole-parent	17.0	13.6 - 20.8
Child's CBCL score - Aggressive & Delinquent (parent and teacher reported)		
Aggressive and Delinquent	2.3	1.5 - 3.3
Either Aggressive or Delinquent	6.8	5.3 - 8.4
Neither Aggressive nor Delinquent	90.9	88.7 - 92.8
Combined carer income		
Low	38.8	34.4 - 43.3
Medium	38.6	34.5 - 43.1
High	22.6	19.6 - 26.2
Region		
Metropolitan	73.5	68.1 - 78.5
Non-Metropolitan	26.5	22.3 - 31.0
Primary carer was a smoker		
Yes	23.8	20.2 - 27.7
Highest school year completed by primary carer		
Year 9 or lower	14.1	11.3 - 17.3
Year 10 or higher	85.9	82.7 - 88.8
Girl's mother was a teenage mother		
Yes	4.4	3.2 - 6.0
Primary carer was in receipt of government benefits		
Yes	36.8	32.7 - 41.1
Importance of religion for child's primary carer		
Very important	21.8	18.7 - 25.3
Reasonably important	34.1	30.7 - 37.5
Not at all important	44.1	40.2 - 48.3
Child was breastfed		
Yes	84.3	81.3 - 86.9
Family functioning		
Poor	10.9	8.7 - 13.7

**Table 2:** Estimated proportion of girls from the WACHS that became pregnant as a teenager, became teenage mothers or who had an abortion as a teenager

	Proportion who became pregnant as a teenager (n = 155)		Proportion who became teenage mothers (n = 94)		Proportion who had an abortion as a teenager (n = 72)	
	*%*	*95% CI*	*%*	*95% CI*	*%*	*95% CI*
**All girls in WACHS (n = 1,374)**	**10.5**	**8.3 - 13.0**	**6.9**	**5.0 - 9.3**	**4.2**	**3.1 - 5.4**

Family type						
Original	7.5	5.6 - 9.6	3.9	2.5 - 5.9	4.0	2.9 - 5.6
Step/blended	16.8	10.5 - 25.7	12.1	7.0 - 19.5	5.5	2.7 - 10.7
Sole-parent	19.6	12.1 - 30.1	17.0	9.6 - 27.8	4.0	2.2 - 7.2
Child's CBCL score - Aggressive &						
Delinquent (parent and teacher reported)						
Aggressive and Delinquent	39.5	19.7 - 61.5	28.0	11.6 - 47.8	11.5	2.5 - 31.2
Either Aggressive or Delinquent	17.5	9.4 - 27.5	13.7	6.6 - 23.2	3.8	0.8 - 11.1
Neither Aggressive nor Delinquent	9.4	7.2 - 12.0	6.0	4.0 - 8.4	4.1	3.1 - 5.5
Child's CBCL score - total t score (parent reported)						
Normal	9.8	7.5 - 12.7	6.3	4.3 - 9.1	4.1	3.0 - 5.4
Abnormal	17.5	9.4 - 27.5	12.1	6.1 - 20.2	5.4	1.0 - 13.1
Not stated	9.4	3.8 - 18.3	7.3	2.7 - 14.9	3.8	0.8 - 11.4
Child's CBCL score - total t score (teacher reported)						
Normal	9.9	7.0 - 13.1	6.3	4.0 - 9.9	4.4	3.1 - 5.9
Abnormal	23.3	14.9 - 34.6	15.8	9.0 - 25.2	7.9	2.4 - 16.3
Not stated	8.1	5.6 - 11.8	5.7	3.5 - 9.0	2.7	1.2 - 5.0
Combined carer income						
Low	15.7	11.2 - 20.8	12.3	8.1 - 17.1	4.3	2.5 - 6.5
Medium	7.3	5.1 - 9.9	3.1	1.8 - 5.0	4.6	2.8 - 6.9
High	6.6	3.9 - 11.0	2.7	0.9 - 6.0	4.3	2.0 - 8.0
Region						
Metropolitan	9.6	6.8 - 12.8	6.8	4.4 - 10.0	3.2	2.1 - 4.8
Non-Metropolitan	12.9	10.2 - 15.8	7.3	5.3 - 9.5	6.9	4.8 - 9.5
Primary carer was a smoker						
Yes	14.6	10.9 - 19.5	10.5	7.2 - 14.7	5.0	2.6 - 8.6
No	9.0	6.5 - 11.9	5.6	3.3 - 8.4	3.9	2.8 - 5.3
Highest school year completed by primary carer						
Year 9 or lower	22.3	12.9 - 33.8	18.5	9.2 - 29.5	5.2	2.6 - 8.9
Year 10 or higher	8.4	6.7 - 10.3	4.9	3.5 - 6.6	4.1	2.9 - 5.4
Girl's mother was a teenage mother						
Yes	24.1	14.4 - 38.4	20.6	11.1 - 34.7	5.1	2.0 - 11.4
No	9.8	7.7 - 12.4	6.2	4.3 - 8.6	4.2	3.1 - 5.5
Primary carer in receipt of government benefits						
Yes	14.9	10.5 - 20.3	12.6	8.2 - 17.7	3.4	1.9 - 5.7
No	7.7	6.0 - 9.8	3.4	2.4 - 4.9	4.7	3.3 - 6.3
Importance of religion for child's primary carer						
Very important	6.3	3.7 - 10.7	4.7	2.5 - 7.9	2.0	0.6 - 5.6
Reasonably important	12.2	7.5 - 18.5	8.9	4.5 - 15.2	3.7	2.2 - 5.6
Not at all important	10.8	6.7 - 25.4	5.8	3.7 - 8.7	6.0	4.0 - 8.8
Child was breastfed						
Yes	10.5	8.2 - 13.3	7.4	5.2 - 10.2	3.7	2.7 - 4.9
No	9.6	5.6 - 15.1	4.1	1.7 - 7.7	6.9	3.3 - 12.1
Family functioning						
Good	8.3	6.3 - 10.5	5.2	3.5 - 7.3	3.8	2.6 - 5.2
Poor	19.5	9.6 - 34.6	13.5	3.6 - 29.8	6.0	2.7 - 11.9
Child's birth weight						
Normal	10.5	8.2 - 13.1	7.2	5.2 - 9.8	3.9	3.0 - 5.1
Low	8.3	3.1 - 17.3	2.9	0.6 - 8.3	6.5	1.7 - 15.0

Nearly 40% of girls who were identified as having both aggressive and delinquent behaviours became pregnant as a teenager, compared with fewer than 10% of girls who had neither of these behaviours. The combination of these behaviours had an increasing effect on both the proportion of girls that became teenage mothers, and the proportion of girls that had an abortion as a teenager.

The proportion of girls who became pregnant and gave birth before age 20 years decreased with increasing combined carer income, while the proportion of girls who had an abortion as a teenager did not vary by combined carer income. Some 12.3% of girls from low income families went on to become teenage mothers, while the proportion for girls from high income families was 2.7%.

Significant differences by family type were noted for those girls who became pregnant as a teenager, and those that became teenage mothers. Girls living in either step/blended or sole-parent families were more likely to become pregnant as a teenager than those living in original families. Some 16.8% of girls who were in a step/blended family at the time of the WACHS went on to become pregnant as a teenager, compared with 7.5% of girls from original families.

Girls were more likely to become pregnant as teenagers if their primary carer was a smoker at the time of the survey (14.6%) compared with those carers that were non-smokers (9.0%). Of those girls whose primary carer completed Year 9 or lower at high school, 22.3% became pregnant as a teenager compared with 8.4% of girls whose carer completed Year 10 or higher at high school. About one-fifth (20.6%) of girls whose mother had been a teenage mother herself went on to become teenage mothers, compared with 6.2% of girls whose mother had not been a teenage mother.

Level of family functioning, whether the teenager was breastfed, importance of religion and birth weight were not significantly associated with the proportion of girls who became teenage mothers, or the proportion that had an abortion as a teenager.

Table 3 shows the results of proportional hazards modelling and reports on the variables associated with risk for teenage pregnancy. It shows that teenage pregnancy was significantly and independently associated with aggressive and delinquent behaviours as reported by primary carers at the time of the survey (HR: 3.52; CI 1.90-6.55); living in a step or blended family type (HR: 2.19; CI 1.41-3.40); living in a family with a low carer combined income (HR: 1.72; CI 1.01-2.92); primary carer completing Year 9 or lower at high school (HR: 1.71; CI 1.14-2.57); primary carer being a smoker (HR: 1.63; CI 1.14-2.31); and living in a non-metropolitan area at the time of the survey (HR: 1.52; CI 1.09-2.11).

**Table 3:** Proportional Hazards Model: Multivariate hazard ratios of associations between characteristics collected during childhood and later teenage pregnancy

*Parameter*	Hazard Ratio	95% CI	p value
Family type			
Original	1.00 (ref)		
Sole-parent	1.10	0.69 - 1.76	0.6934
Step/blended	2.19	1.41 - 3.40	<0.001
CBCL score (child) - Aggressive and			
Delinquent			
Neither	1.00 (ref)		
Aggressive and Delinquent	3.52	1.90 - 6.55	<0.001
Aggressive or Delinquent	1.24	0.71 - 2.14	0.448
Combined carer income			
High	1.00 (ref)		
Medium	0.93	0.55 - 1.58	0.794
Low	1.72	1.01 - 2.92	0.045
Highest school year completed by primary carer			
Year 10 or higher	1.00 (ref)		
Year 9 or lower	1.71	1.14 - 2.57	0.009
Region			
Metropolitan	1.00 (ref)		
Non-Metropolitan	1.52	1.09 - 2.11	0.014
Whether primary carer was a smoker			
No	1.00 (ref)		
Yes	1.63	1.14 - 2.31	0.007

Teenage pregnancy was not independently and significantly associated with whether the girl's mother had been a teenage mother, level of family functioning, size of the family, whether the girl's primary carer had ever been treated for an emotional or mental health problem, SEIFA [29], type of school the girl had attended, how well the girl did at school (as reported by either the girl's primary carer or teacher), whether the girl needed professional help for emotional or behavioural problems (as reported by the primary carer), primary carer's marital status, primary carer's satisfaction level with the girl's progress in her education and learning skills, level of importance of religion to the girl's primary carer and whether the girl was breastfed.

**Figure 1:**
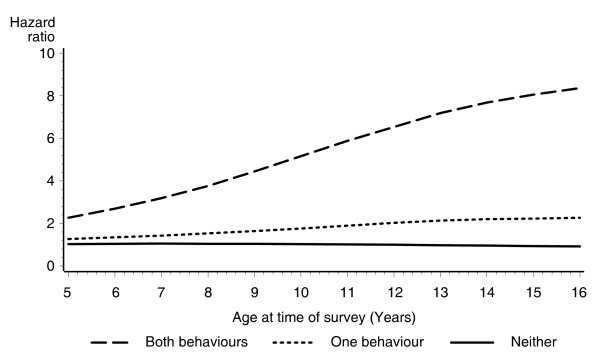
**Unadjusted hazard ratios for teenage pregnancy, for children rated as having both aggressive and delinquent behaviours, one of these behaviours or neither of these behaviours, by age of child when factor was measured**.

**Figure 2:**
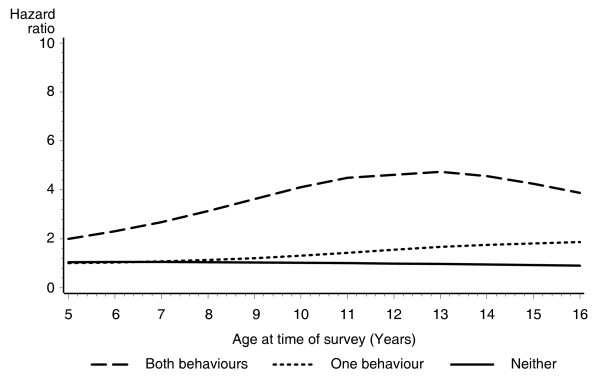
**Adjusted hazard ratios for teenage pregnancy, for children rated as having both aggressive and delinquent behaviours, one of these behaviours or neither of these behaviours, by age of child when factor was measured**.

To investigate whether age of the girl at the time of the survey affected the association between aggressive and delinquent behaviours and risk for teenage pregnancy, two age interaction models were fitted. Figure 1 shows the age interaction model which is unadjusted for other factors, while Figure 2 is adjusted for the other significant variables in the proportional hazards model (family type, combined carer income, highest school year completed by the girl's primary carer, whether the primary carer was a smoker and whether the girl lived in a metropolitan or non-metropolitan area at the time of the survey). Figure 1 shows that if a girl exhibited both aggressive and delinquent behaviours, she was between two to eight times as likely to become pregnant as a teenager compared with girls who showed neither of these problems. After adjusting for the other factors, Figure 2 showed that if a girl exhibited both aggressive and delinquent behaviours, she was between two to four times as likely to become pregnant as a teenager compared with girls who showed neither of these problems. The hazard curve was significantly elevated for girls who exhibited both aggressive and delinquent behaviours (χ^2 ^= 12.4; *p *< 0.001), but there was no significant difference between the hazard for girls who exhibited one of these problems compared with girls who showed neither (χ^2 ^= 1.53; *p *< 0.448).

## Discussion

Of the eight mental health problems measured by the Child Behavior Checklist [25], two were strongly associated with teenage pregnancy - delinquent behaviour (including breaking rules and norms set by parents and communities) and aggressive behaviour (including bullying, teasing, temper tantrums, arguing, fighting and threatening behaviours). While many of the factors assessed in the survey would be expected to be reasonably constant over time, such as carer education, one factor that may well vary depending on the age of the child at the time of the survey is aggressive and delinquent behaviours. One might expect that the relationship with pregnancy outcomes would be strongest for girls who were older at the time of the survey. For example, knowing that a 16 year-old is aggressive and delinquent might be expected to be a stronger predictor of teenage pregnancy than observing the same behavioural problems in a 4 year-old. Some young children with behavioural problems will grow out of these problems by their teenage years, while for other children the onset of these problems would be at older ages. To address this issue, we fitted an age interaction with aggressive and delinquent behaviours. The adjusted association with aggressive and delinquent behaviours was strongest among girls aged 11 years and over, however the presence of the combination of these two mental health problems was significantly associated with teenage pregnancy regardless of the age at which it was identified. While other studies have identified that delinquent teenagers often proceed to risk behaviours such as drug taking and early sexual initiation [33,34], our study extends other findings that found an association between conduct problems in girls aged 8 years and later teenage pregnancy [35].

Previous literature has shown that children of teenage mothers are more likely to exhibit aggressive and delinquent behaviours. One study found that adolescent children of younger mothers (aged 18-years or younger at the time of their first antenatal visit) were more likely to be delinquent and aggressive than the children of older mothers [36]. Another study suggested that the young children of teenagers were more likely to have higher levels of aggression than the young children of older mothers [13]. Together the studies suggest that the daughters of teenage mothers are more likely to show aggressive and delinquent behaviours, while we have found that these behaviours increase the probability that they too will become teenage mothers. Other studies suggest that teenagers who exhibit aggressive and delinquent behaviours are more likely to engage in sexual risk behaviours, including those that result in teenage pregnancy [33,37]. It is important to prevent and manage aggressive and delinquent behaviours early before they become entrenched, but additionally, our results suggest that addressing these behaviours as early in developmental pathway as possible may reduce the likelihood of teenage pregnancy.

Whether the girl's mother was herself a teenage mother was strongly associated with teenage pregnancy outcomes, but was not a significant independent predictor in the final model. This suggests that other factors included in the final model, such as family type, income and education level, and carer smoking mediate the relationship in teenage motherhood from one generation to the next. The circumstances of a teenage mother are often low education, poverty and difficult family circumstances. Once we accounted for these factors, the association is not strong enough to remain independently significant.

Primary carers who were smokers were more likely to have daughters who became pregnant as teenagers. Smoking by primary carers remained a significant predictor of teenage pregnancy even after adjusting for a range of other variables including socio-economic status as measured by SEIFA [29], highest school year completed by primary carer, family type, primary carer in receipt of government benefits, combined carer income and whether the teenager lived in a metropolitan or non-metropolitan area as a child. Despite the presence of a wide range of other demographic and psycho-social variables, it is possible that smoking status was masking another variable that was not collected in our original survey and was not available via the data linkage. One variable that we could not account for adequately was mental health of the carer, and there is an established association between mental health problems and smoking [38,39]. This issue deserves further investigation. When carer smoking status was assessed in 1993, smoking rates were higher than they are now. Nevertheless the health consequences of smoking had been well publicised for many years at that stage, and it is quite likely that carers, principally mothers, who were smokers were systematically different from non-smokers in ways other than just the demographic and psycho-social variables measures in the survey.

Girls from step/blended families were significantly more likely than girls from original families to become pregnant as teenagers. The difference between sole-parent families and original families was significant in the bivariate analysis but not significant in the adjusted proportional hazards regression model. Our data do not allow us to speculate as to whether the higher teenage pregnancy rate was impacted by the original family breaking up, or the arrival of the new parent in the step/blended family, or a combination of these two issues. We did not know the girl's age at the time of the family break up, or her age at the time of the arrival of the step parent. Others have also found an effect of family type on rates of young motherhood [10,24,40,41]. Girls are more likely to delay first sexual intercourse and use contraception at first intercourse if they had lived with both natural parents to sixteen years of age [42]. Sole parent and step/blended family status are associated with higher risks for aggression and delinquency in young people [43]. Girls from step/blended families had an elevated risk for teenage pregnancy after adjusting for education and income factors.

Combined carer income and the highest year of school completed by the primary carer had strong effects on predicting risk for teenage pregnancy, even after controlling for a host of other child specific and family variables. The daughters of families with a low combined carer income, and those of carers who completed Year 9 or lower at high school, were more likely to become pregnant as teenagers, findings which were consistent with other research [34,36,42,44]. Individual or household based socioeconomic measures such as these were stronger predictors of teenage pregnancy than community based measures such as SEIFA [29]. In addition, one study found that girls from socioeconomically disadvantaged households tended to know less about sex and contraception, and to have negative or indecisive attitudes regarding condom use, increasing their risk of becoming pregnant as teenagers [45].

A major strength of this study is the increased analytic power which comes from linking data from our 1993 cross sectional survey to administrative data collected in the fourteen years that followed. An additional strength is the wide range of variables, based on a social-ecology model, available from the WACHS. With respect to the data on abortions of teenage pregnancies, there is evidence to suggest that self-reported data on abortions may lead to under-reporting [46-48]. Our data linkage design avoids the bias associated with self reports and provides an opportunity to obtain accurate and less biased data on abortions. Another strength of our study is the high linkage rate of the 2,304 WACHS children that were born in WA, 2,282 were linked to their birth records. Of the 432 WACHS children that were born outside WA, 355 linked to the morbidity, mental health or electoral roll records. The overall level of inaccuracies in the WADLS (including false positives and false negatives) has been estimated at 0.11% [49].

This study has several limitations. Data on pregnancies includes only abortions and live births (i.e. miscarriages have been excluded). We did not have complete ascertainment of abortions, as data from some private clinics was not available via the data linkage. We used the actual abortion proportion for WA to confirm that the numbers we had observed in our population were close to what was expected. The abortion proportion is derived by taking the number of abortions divided by the number of abortions and live births combined. The average abortion proportion in WA over 2002-2005 for women aged less than 20 years was 51.9% [50]. For the teenagers in our study, we had 72 abortions and 94 live births, an abortion proportion of 43.4%.

It is inevitable that some girls sampled in the WACHS had moved out of WA since 1993, and therefore had reduced opportunity to have a record on the administrative datasets that were used in the data linkage. However, analysis of the number of survey children now on the electoral roll in WA showed that approximately 86% had enrolled to vote in WA, suggesting that the capture rate was still high.

With the available sample size, the study may not have had sufficient power to detect associations with risk factors that occurred rarely or were associated with small effect sizes. It is thus possible that some of the risk factors we considered but did not find significantly associated with teenage pregnancy have associations that were too small for the power of our study to detect.

Finally, it may have been useful to analyse the CBCL scores from the teacher and parent reports separately, in particular, those children who were rated as aggressive and delinquent. One study suggested that parent and teacher reports of child behaviours are not always linked, meaning that often only one source reports a problem while the other does not [51]. Although there was not sufficient statistical power to separate the parent and teacher reports of child behaviours, both showed similar patterns.

## Conclusions

It is known that girls who become pregnant as a teenager will face increased medical risks, and if the pregnancy is taken to term, there are also increased medical risks for their babies. We also know that teenage mothers, and their children, can suffer from poorer life outcomes. In addition, the children of teenage mothers are more likely to become teenage mothers, perpetuating the intergenerational cycle of teenage pregnancies. With these consequences in mind, we should aim to further reduce the incidence of teenage pregnancy, in particular those pregnancies that are unwanted and unintended.

At the outset, we hypothesised that there are factors in early life that are potential predictors of teenage pregnancy. Our findings have confirmed this, and knowledge of these predictive factors should be used to inform future preventive policy interventions. Our findings suggest that interventions to reduce teenage pregnancy rates could address other issues in addition to the traditional focus on sex education programmes. In particular, early intervention to treat behavioural problems may also be useful in reducing the incidence of unwanted teenage pregnancy.

## Competing interests

The authors declare that they have no competing interests.

## Authors' contributions

SZ and SS undertook the 1993 WA Child Health Survey. SZ, SS and FJS conceived the original idea for this data linkage study. All authors contributed to the development of the study methodology. JG undertook the data analysis and wrote the first draft of the manuscript, with assistance from DL and FM. All authors edited the paper. All authors read and approved the final manuscript.

## Pre-publication history

The pre-publication history for this paper can be accessed here:

http://www.biomedcentral.com/1471-2458/10/63/prepub
